# 3D Printed Porous Methacrylate/Silica Hybrid Scaffold for Bone Substitution

**DOI:** 10.1002/adhm.202100117

**Published:** 2021-05-05

**Authors:** Justin J. Chung, Jin Yoo, Brian S. T. Sum, Siwei Li, Soojin Lee, Tae Hee Kim, Zhenlun Li, Molly M. Stevens, Theoni K. Georgiou, Youngmee Jung, Julian R. Jones

**Affiliations:** Department of Materials, Imperial College London London SW7 2AZ, United Kingdom; Center for Biomaterials, Biomedical Research Institute, Korea Institute of Science and Technology (KIST), Seoul 02792, Republic of Korea; Center for Biomaterials, Biomedical Research Institute, Korea Institute of Science and Technology (KIST), Seoul 02792, Republic of Korea; Department of Materials, Imperial College London London SW7 2AZ, United Kingdom; Department of Materials Imperial College London London SW7 2AZ, United Kingdom; Center for Biomaterials, Biomedical Research Institute, Korea Institute of Science and Technology (KIST), Seoul 02792, Republic of Korea; Center for Biomaterials, Biomedical Research Institute, Korea Institute of Science and Technology (KIST), Seoul 02792, Republic of Korea; Department of Materials Imperial College London London SW7 2AZ, United Kingdom; Department of Materials Imperial College London London SW7 2AZ, United Kingdom; Department of Materials Imperial College London London SW7 2AZ, United Kingdom; School of Electrical and Electronic Engineering, Yonsei University, Seoul 03722, Republic of Korea; Center for Biomaterials, Biomedical Research Institute, Korea Institute of Science and Technology (KIST), Seoul 02792, Republic of Korea; YU-KIST Institute, Yonsei University, Seoul 03722, Republic of Korea; Department of Materials Imperial College London London SW7 2AZ, United Kingdom; Institute of Biomedical Engineering, Imperial College London, London SW7 2AZ, United Kingdom; Department of Bioengineering, Imperial College London, London SW7 2AZ, United Kingdom

**Keywords:** 3D printing, biomaterials, bone substitutes, hybrids, sol-gels

## Abstract

Inorganic–organic hybrid biomaterials made with star polymer poly(methyl methacrylate-*co*-3-(trimethoxysilyl)propyl methacrylate) and silica, which show promising mechanical properties, are 3D printed as bone substitutes for the first time, by direct ink writing of the sol. Three different inorganic:organic ratios of poly(methyl methacrylate-*co*-3-(trimethoxysilyl)propyl methacrylate)-*star*-SiO_2_ hybrid inks are printed with pore channels in the range of 100-200 μm. Mechanical properties of the 3D printed scaffolds fall within the range of trabecular bone, and MC3T3 pre-osteoblast cells are able to adhere to the scaffolds in vitro, regardless of their compositions. Osteogenic and angiogenic properties of the hybrid scaffolds are shown using a rat calvarial defect model. Hybrid scaffolds with 40:60 inorganic:organic composition are able to instigate new vascularized bone formation within its pore channels and polarize macrophages toward M2 phenotype. 3D printing inorganic-organic hybrids with sophisticated polymer structure opens up possibilities to produce novel bone graft materials.

## 1 Introduction

There is currently an unmet clinical need for medical devices that can help heal non-union bone fractures, which are damaged bones that cannot heal on their own or under conventional treatment. Metal implants can be used but are often too stiff, potentially causing bone loss due to stress shielding,^[1]^ or not transmitting mechanical cues to osteogenic cells, or they become encapsulated by fibrous tissue, leading to micromotion.^[2]^ Bioactive ceramics (e.g., synthetic hydroxyapatite) and bioactive glasses (e.g., Bioglass) can bond directly to bone, and stimulate high quality bone growth,^[3]^ but they are stiff and brittle.^[4,5]^ Inorganic–organic sol-gel hybrids are of great interest for bone repair and have shown potential to surpass bioactive glass scaffolds due to tailorable mechanical properties, such as the ability to withstand cyclic loading.^[6,7]^ These properties stem from the nanoscale interlocking of inorganic and organic co-networks and covalent bonds between the co-networks.^[8,9]^ Inorganic–organic hybrids have been previously produced via the sol-gel process, using natural polymers functionalized with an organosilane coupling agent, such as glycidoxypropyl trimethoxysilane (GPTMS),^[10–13]^ to enable covalent bonding between the co-networks. However, controlling the chemical reaction between the coupling agent, for example, the epoxy ring of GPTMS to nucleophiles on the polymer chain, is difficult.^[14]^ Natural polymers also come with the challenge of whether there is a reproducible source. Synthetic polymers are much more versatile for hybrid synthesis. Copolymers of methyl methacrylate (MMA) and 3-(trimethoxysilyl)propyl methacrylate (TMSPMA) have shown excellent potential as an organic source, as TMSPMA can form bonds between the organic and silica co-networks,^[15,16]^ and the copolymer-silica hybrids have shown biocompatibility in vivo^[17]^ and in vitro.^[18]^ Copolymers of poly(MMA-*co*-TMSPMA) with different architectures enabled synthesis of hybrids with tailorable mechanical properties, for example, star polymer architectures, which resemble flexible star gels,^[19]^ within which the arms and crosslinking core can be modified independently.^[20,21]^ Until now, the poly(MMA-*co*-TMSPMA) hybrids have not been made into porous scaffolds suitable for bone repair. An ideal bone scaffold must have an open pore network with interconnections between the pores in excess of 100 μm to allow a passageway for vascularized bone ingrowth.^[4,22]^ 3D printing is advantageous over foaming,^[12]^ electro-spinning,^[23]^ and freeze drying^[10]^ techniques, because the pores and their inter-connects can be precisely controlled.^[24]^ If 3D grid-like structure printing is possible, the channels can be wide open while the scaffold can have the compressive strength of bone.^[25]^ An advantage of using the sol-gel process is that the hybrid sol can be printed directly, in a layer-by-layer extrusion printing process.^[7]^ Previously, 3D printed silica/poly-tetrahydrofuran/poly-*ε*-caprolactone (SiO_2_- pTHF-PCL) hybrid scaffolds were found to provoke human bone marrow derived stem cells down a chondrogenic route, when pore channel sizes were ≈250 μm.^[26]^ They were also able to withstand cyclic loads, and even self-heal. However, control of the “ink” gelation and identifying a “printing window” (time within which the ink can be printed but also hold its own weight) are challenging.

Here, printability of poly(MMA-*co*-TMSPMA)-*star*-SiO_2_ hybrid formulations, developed in a previous study,^[20]^ was investigated. The stars consisted of linear poly(MMA-*co*-TMSPMA) arms with a molar ratio of MMA_100_-TMSPMA_10_ linked with an ethylene glycol dimethacrylate (EGDMA) core. The effect of different inorganic:organic ratios (in wt%); 50:50, 40:60, and 30:70, of the 3D printed hybrids on bone repair in vivo were examined.

## 2 Results and Discussion

### 2.1 Hybrid Confirmation

**Figure 1:**
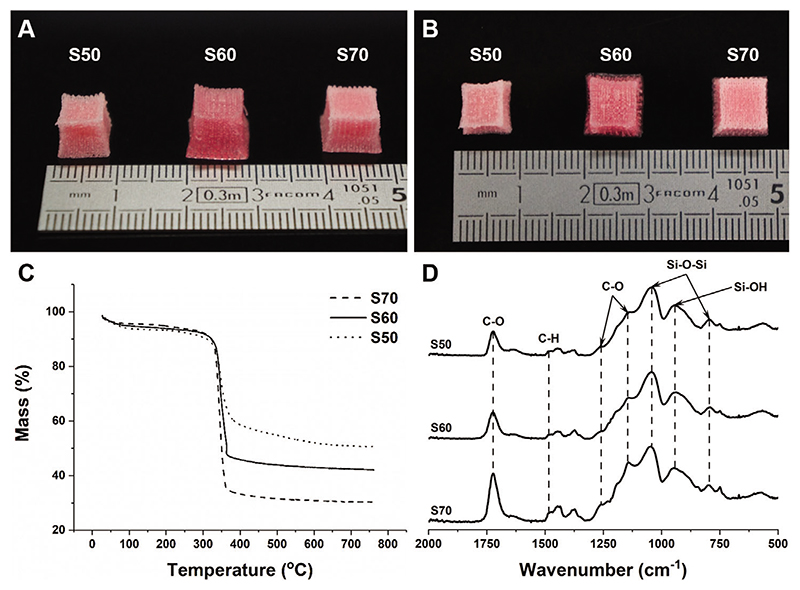
A) Side, and B) top photographs of 3D printed hybrid scaffolds of different inorganic:organic compositions (the numbers in S50, S60, and S70 refer to organic wt%). C) TGA curves and D) FTIR spectra of the hybrid scaffolds made with different organic contents, containing: absorption bands of organic polymer (i.e., C–H vibration, C=O, C–C–O asymmetric, and C–O–C symmetric stretch) and of the condensed silica network (i.e., Si–O–Si asymmetric and Si–OH stretch).

Poly(MMA-*co*-TMSPMA)-*star* copolymer was synthesized through reversible addition-fragmentation chain-transfer (RAFT) polymerization technique with the arm-first approach, and then it was mixed with hydrolyzed silica network precursor (tetraethyl orthosilicate, TEOS) in three different inorganic:organic ratios of 50:50, 40:60, and 30:70. Hybrid scaffolds were 3D printed by direct ink writing and the compositions will be referred to as S50, S60, and S70 (the number corresponds to the organic content wt%, Figure 1A,B). The pink coloration of the scaffolds was from dithiol bonds of the RAFT agent used. In the previous study, hybrid monolith cylinders were made successfully only when the inorganic content was 30 wt% or lower as higher inorganic content was susceptible to cracking. This was due to shrinkage during drying and capillary stresses causing fracture.^[9]^ Here, capillary stress was reduced through 3D printing, resulting in a higher surface area to volume ratio of the scaffold structure (thin struts rather than a bulk material).^[27]^ The actual inorganic:organic ratios were confirmed by thermogravimetry (TGA, Figure 1C). All the hybrid scaffolds showed thermal decomposition at 360°C, due to oxidation of the polymer.^[16,20,28]^ Residual mass remaining from S50, S60, and S70 were close to our targeted inorganic content of 50.5%, 42.1%, and 30.2% respectively. Fourier transform infrared spectroscopy (FTIR) was conducted to confirm the scaffolds’ molecular structure (Figure 1D). The FTIR spectra of the scaffolds were similar to those of monoliths produced previously.^[20]^ The TGA and FTIR results confirmed that both polymer and silica network were present in the scaffolds.

### 2.2 3D Printing Process and Scaffold Characterization

3D printing inorganic-organic hybrids with consistent quality was a challenging process.Height of the 3D printing syringes and their nozzles varied (± ≈1-2 mm), due to variation in supply from the manufacturer, therefore position of the ink syringe had to be adjusted every printing session. An ideal gap between the syringe nozzle to the printing platform was 0.2 mm. Due to the fluid to viscous (sol to gel) transition during the gelation process, there was a printing window, defined as the time period after mixing the polymer and the sol, when hybrid ink was viscous enough to retain its shape after printing but still fluid enough to be extruded from the nozzle.^[29]^ Additionally, viscosity of the hybrid ink gradually increased during printing, due to covalent bonding formation between polymer to silica network and solvent evaporation (i.e., ethanol, THF, and trace of water). The printing window for all the compositions tested in this study was 1h, but decreased as organic content increased (i.e., S70 gelled faster than other compositions).

**Figure 2:**
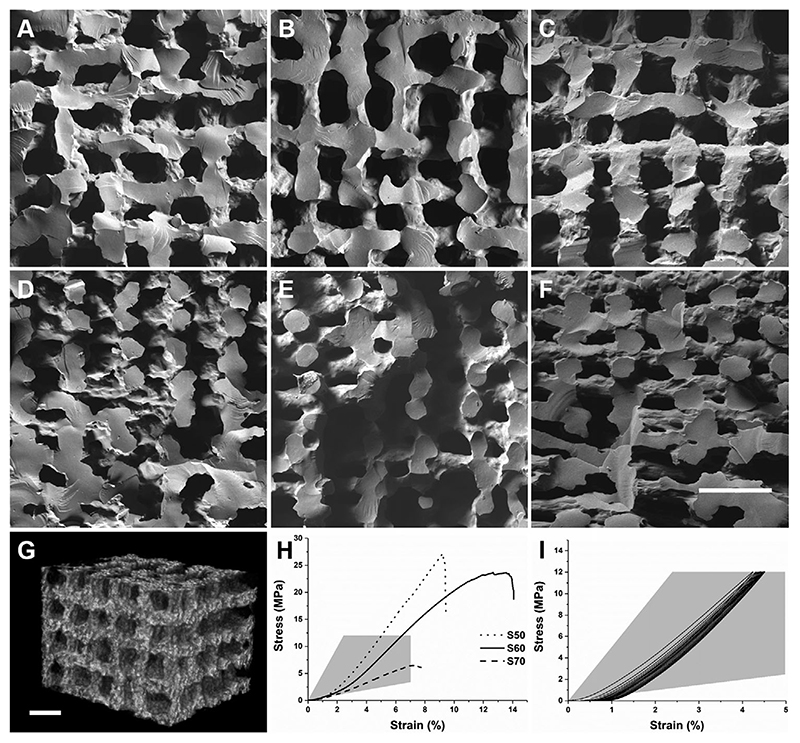
SEM images of horizontal cross-sections of 3D printed hybrid scaffolds A) S50, B) S60, and C) S70 (the numbers in S50, S60, and S70 refer to organic wt%). SEM images of vertical cross-sections of D) S50, E) S60, and F) S70. G) Representative 3D micro-CT image of the central region of S60 (scale bars: 500 μm). H) Representative uniaxial stress/strain curves of the 3D printed scaffolds. I) Cyclic compressive loading stress/strain curve of S60 (mechanical property region of trabecular bone^[1,32]^ is highlighted in grey).

Scanning electron microscope (SEM) images of the horizontal and vertical cross-sections of the scaffolds are shown in Figure 2. The actual inter-strut distance was narrower than the printed distance due to the shrinkage. The shrinkage resulted from the evaporation of solvents during drying stage as well as polymerization of the hybrid from the condensation reaction.^[13]^ An ideal inter-strut distance was determined by printing S60 hybrids with different inter-strut dimensions. Printed scaffolds were optimal when printed with a pore size between 100 and 200 μm. A pore size of 500 μm, which is in the range of trabecular bone pore sizes, was produced with a nominal 1 mm inter-strut distance. However, as shown in Figure S1A,B, Supporting Information, the struts were sagging between underlying struts and collapsing in the *z* direction; resulting in poor interconnectivity. A nominal inter-strut distance of 0.7 mm also produced horizontally merged and collapsed porous structure, despite having vertical pore size of ≈300-500 μm (Figure S1C,D, Supporting Information). 3D printing with 0.5 mm inter-strut distance enabled fabrication of scaffolds with improved interconnected porous structure in both horizontal and vertical directions (Figure 2). Hence, hybrid scaffolds with different compositions were printed with 0.2 mm printing nozzle (strut diameter) and 0.5 mm inter-strut distance (pore size). After drying, the strut diameter shrank to ≈100 μm and pore size was between 100 and 200 μm (Table 1), meeting the hypothesized minimal requirements for vascularization.^[4,30]^ The horizontal cross-section SEM images (Figure 2A,B,C) displayed vertical channels running through the scaffolds with ≈190 μm widths for S70 and S60 scaffolds. S50, on the other hand, had pore width of 180 μm, as scaffolds with the higher inorganic content were more susceptible to shrinkage. The vertical cross-section images (Figure 2D,E,F) also showed inter-connectivity of pores and bonding between the struts of each layer. However, lower pore sizes were observed compared to the horizontal cross-section. S70 had an average pore size of 111 μm, which is due to higher organic content, resulting in less stiff ink that compressed under its own weight while printing. S50 also showed merging of the layers producing average pore size of 121 μm; possibly caused by higher inorganic content inhibiting to gel. From the SEM images, S60 had the most defined porous structure. The pore interconnectivity and porosity of S60 was confirmed geometrically, using helium pycnometry to obtain the skeletal density. The percent porosity was 60.5 ± 1.1%, which is within the range of trabecular bone.^[31]^ The qualitative pore inter-connectivity was confirmed through micro-CT scans. The reconstructed 3D image of the central region (Figure 2G) and volumetric rendering of S60 (Video S1, Supporting Information) also showed inter-connected porous structure.

Uniaxial compression tests were performed. An ideal bone substitute should have mechanical properties that are similar to that of bone.^[1]^The stress-strain curves (Figure 2H) indicate that all the scaffolds are within the range of trabecular bone, which is reported to have Young’s modulus values in the range of 50-500 MPa and compressive strength of 2-12 MPa (shaded region in Figure 2H).^[1,32]^ The general trend of Young’s modulus and failure stress of the scaffolds increased as inorganic content of the hybrids increased, which agrees with previous studies on poly(MMA)-silica hybrids.^[33]^ As shown in Table 1, S50 had the highest failure stress of 26.3 MPa at a strain to failure of 8.9%. S70 had the lowest failure stress which was more than threefold lower than other compositions, while failure strain value was 7.4%. S60 had the most synergistic properties, that is, flexibility from polymer and strength from silica matrix, with the highest strain to failure of 11.5%, failure stress of 22.4 MPa and Young’s modulus of 247 MPa. Cyclic compressive loading tests were also performed on S60 to confirm ductility for 12 MPa compressive stress, which is at the upper end of the compressive strength range of trabecular bone. As Figure 2I shows, the scaffold did not appear to fracture and the only noticeable hysteresis was observed during the first cycle, which is common in such tests, possibly from an uneven top/bottom surface of the scaffold. Cyclic deformations decreased after each cycle, which was less than total of 0.5% strain stretch.

The mechanical properties of the scaffolds not only meet those required of trabecular bone, but also surpassed previous 3D printed bone substitutes. In terms of previously printed hybrid scaffolds, hybrids with methacrylic copolymers have been 3D printed previously,^[27]^ but their pore channel size was 2 mm and mechanical properties were only extrapolated from three point bending tests of bulk samples. Gelatin-SiO_2_ hybrid with 25 wt% gelatin was 3D printed with pore channel size of ≈550 μm and low porosity (30%), perhaps due to high shrinkage.^[34]^ Despite having higher inorganic content and denser porous structure, the scaffold had compressive strength of ≈5 MPa with failure strain of ≈5% (estimated from published stress/strain graphs^[34]^). SiO_2_-pTHF-PCL hybrids, with 75 wt% organic component, was printed with a pore channel size of 200 μm (42% porosity) and were highly elastic, with a 36% strain to failure at a compressive strength of 1.2 MPa, which was seen as ideal for cartilage regeneration but too flexible for bone repair.^[7]^

### 2.3 Cytotoxicity and Pre-Osteoblast Adherence Evaluations on the Hybrid Scaffolds

**Figure 3:**
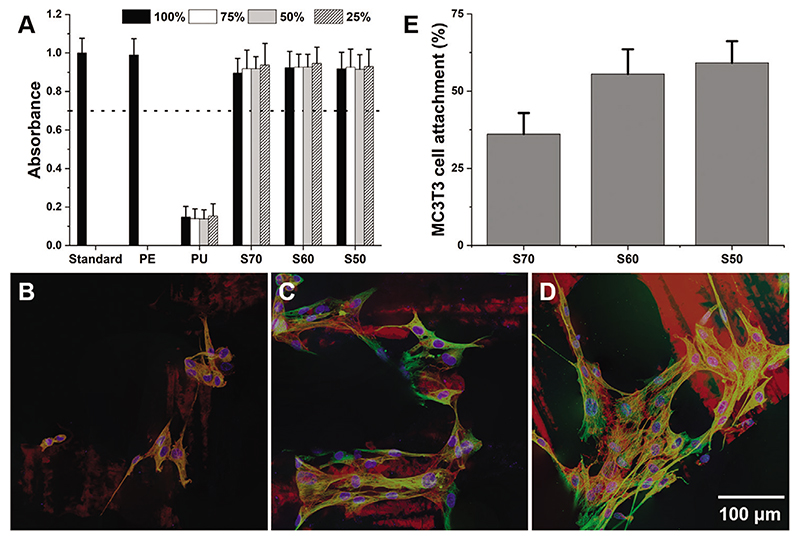
A) MTT metabolic activity assay performed in accordance to ISO 10993: MC3T3 cells were cultured with dissolution products of the hybrids and results were normalized against basal culture medium (Standard). PE refers to media conditioned with the non-toxic negative control material and Polyurethane (PU) was a positive control (for toxicity). Based on the ISO guidance, all test materials (S70, S60, and S50) in the current study can be deemed non-cytotoxic/biocompatible, as the viability of cells is >70% of non-cytotoxic controls (marked by the dashed line). Immunohistochemical staining of MC3T3 cells on B) S70, C) S60, and D) S50. Images were produced by stacks of vimentin immunostain (green), f-actin labeling (red), and DAPI nuclear counter stain via confocal microscopy. E) Percentage of seeded MC3T3 cells attachment on the scaffolds determined using fluorescent Hoechst stain.

For an ideal scaffold for bone repair, the scaffold material must also be non-cytotoxic and promote osteogenic cell adherence.^[35]^ Cytotoxicity of the scaffolds were evaluated by ISO 10993-12.^[36]^ MTT metabolic assay (Figure 3A) confirmed that all the scaffolds passed ISO standards for cytotoxicity, with viability of the cells exposed to extractables of the hybrid scaffolds at >87% to that of the control media, which proved that most of the solvents were removed by drying. The MC3T3 pre-osteoblasts were able to adhere on the scaffolds within 24 h. As shown in Figure 3B,C,D, major cytoskeletal constituents of intermediate filaments and microfilaments were observed along with DAPI staining of nuclei. Although methacrylic polymers are known to be hydrophobic, MC3T3 cells were able to attach on S70 which had the highest amount of polymer. This was expected since monolithic hybrids with the same composition have shown cell attachment previously.^[6,20]^ Total DNA quantification of MC3T3 cells on each scaffold (Hoechst stain) (Figure 3E) revealed that the scaffolds had cell attachment rate of 59 ± 7%, 56 ± 8%, and 36 ± 7% for S50, S60, and S70, respectively (Table 1). The improvement in cell adherence as inorganic content increased was possibly due to increase in stiffness and improvement in hydrophilicity, due to surface silanol groups, which corroborates with a previous study on hybrid monoliths with a range of degree of hydrophilicity.^[6]^

### 2.4 S60 Hybrid Scaffold as a Bone Substitute

**Figure 4:**
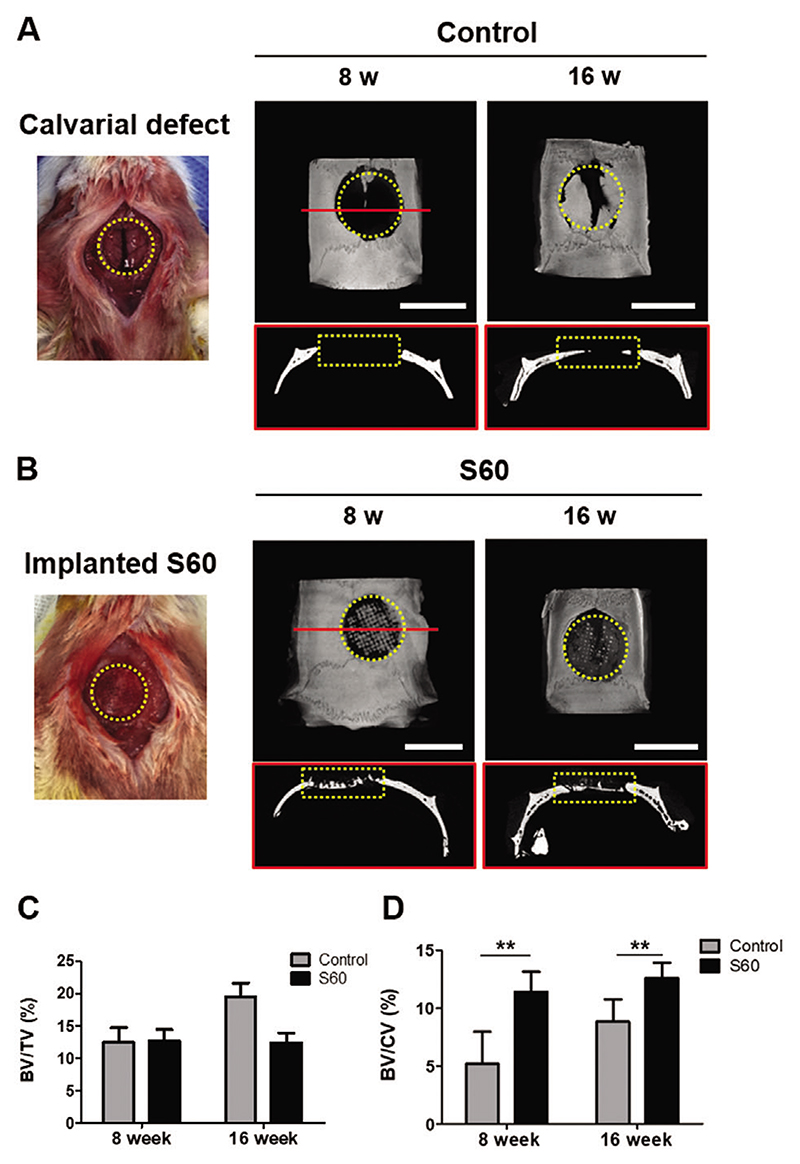
A) Representative photograph of critical size calvarial defect *(φ* 8 mm highlighted with yellow dotted line) model in a mouse, and *μCT* images ofthe cranial bone defects: 3D and coronal (2D slice along the red line of 3D image) at 8 and 16 weeks. B) Representative photograph of implantation of S60 scaffold in the calvarial defect, and *μCT* images of S60 implanted calvarial defects at 8 and 16 weeks (scale bars: 8 mm): 3D and coronal *μCT* images. All the in vivo experiments were conducted on six animals. C) Morphometric analysis of the volume of newly formed bone volume (BV/TV) relative to total skull defect volume (8 mm-sized disk defect). D) Ratio of newly formed bone volume to central volume (5 mm-sized disk defect) (BV/CV) calculated by CTAn program (*n* = 4, **P < 0.01).

The osteogenic properties of S60 were evaluated through a calvarial defect model, since: printability of S60 was superior to other compositions; its material interface was pre-osteoblast friendly; and its mechanical properties were comparable to trabecular bone. A critical sized defect (diameter *(φ):* 8 mm) rat calvarial model was used.^[37]^ Representative micro-computed tomography (*μ*CT) images are shown in Figure 4. The images include 3D reconstructions and 2D cross section slices through the center of the defects. Defects containing S60 implants were compared to the defects without scaffolds (control group). Reconstructed 3D *μCT* images after 8 and 16 weeks post-surgery showed new bone formation in both the control group and defect model with implanted S60 (Figure 4A,B). At 8 weeks, new bone formation in the control group was small, and it started from edges of the host bone, while 2D slice across the center of the defect (coronal section) showed no bone ingrowth. More new bone tissue intruded into the porous structure of S60, and bone ingrowth along the interface of the scaffold-bone was confirmed. At 16 weeks, bone was present in both the control defect and in defect containing scaffold. The quantitative analysis of *μCT* images indicated that the percentage of new bone volume relative to total defect volume (BV/TV) in the S60 group was 12.4%, which was lower than the control group of 19.6% (Figure 4C). This was likely due to new bone formation in the defect interface of bony borders in the control group while the scaffold took up space in the defect site. The defect with S60 implant displayed both peripheral and central distribution of new bone formation. Since the innate healing capacity that originates from the defect margin is known to be constant regardless of the defect size,^[38]^ osteogenic evaluation of the central area of a defect is an important factor in criticalsized bone defect models. Following a previous study on evaluating bone regeneration^[39]^ bone formation of the central area was evaluated by the BV to central volume, that is, *φ* 5 mm within *φ* 8 mm defect (Figure 4D). S60 was able to enhance bone formation compared to that of the control model in both 8 and 16 week post surgeries. The 3D porous structure with osteoblast friendly surface^[40]^ possibly allowed more suitable environment for bone formation.

**Figure 5:**
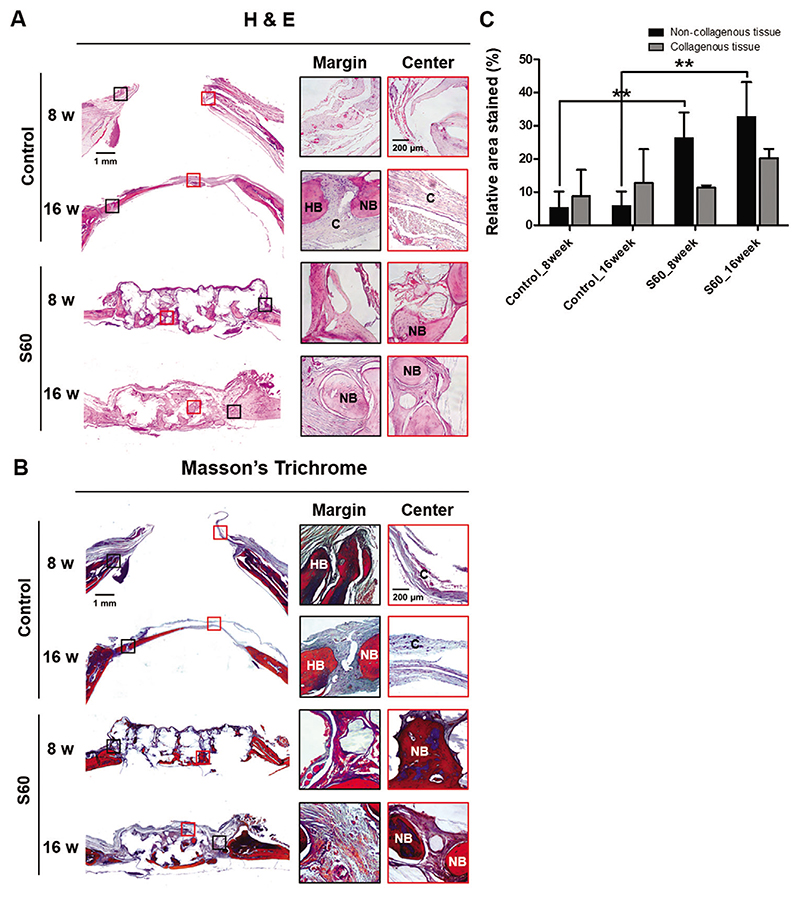
Histological evaluation of calvarial bone regeneration: A) Hematoxylin and eosin (H&E) and B) Masson’s trichrome (MT) staining of calvarial bone defects in control and S60 groups at 8 and 16 weeks after implantation (black and red inset boxes in the left panels indicate the areas shown in the enlarged images of the edge and center of the defect, respectively. C: connective tissue; HB: host bone; NB: new bone). All the in vivo experiments were conducted on six animals. C) Quantitative analysis of the mature bone tissue formation based on MT staining images. The black and grey bars represent the area of bone matrix and collagenous regions, respectively (*n* = 4, **P < 0.01).

Results from the *μCT* analysis were further confirmed by histological assessment. Figure 5A,B shows the histological images of the specimens at 8 and 16 weeks of post implantation. Hematoxylin and eosin (H & E) and Masson’s trichrome (MT) staining were utilized to further evaluate the bone regeneration patterns within S60. The control group showed bone in-growth pattern only from the peripheral region, while S60 groups revealed bone regeneration from the peripheral region plus in situ bone islands formation in the center of defect. At 16 weeks, thin connective tissue formation was detected in control defects, whereas bone-like tissue formation was observed in the areas grafted with S60. Substantial soft connective tissue, stained as blue color in MT staining, was observed in defects of the control group. On the other hand, red colored staining, representing matured bone, was apparent in the central region of the defects after 16 weeks of S60 implantation. Based on the MT staining, mature bone tissue formation was quantified through measuring non-collagenous tissue region; area with red staining, of the central defect area *(φ* 5 mm within *φ* 8 mm defect) (Figure 5C). More matured bone was present for defects with S60 compared to the defects of control group at 8 and 16 weeks post implantation. These histology quantification results corroborated with the bone volume to central volume (BV/CV) *μCT* results.

**Figure 6:**
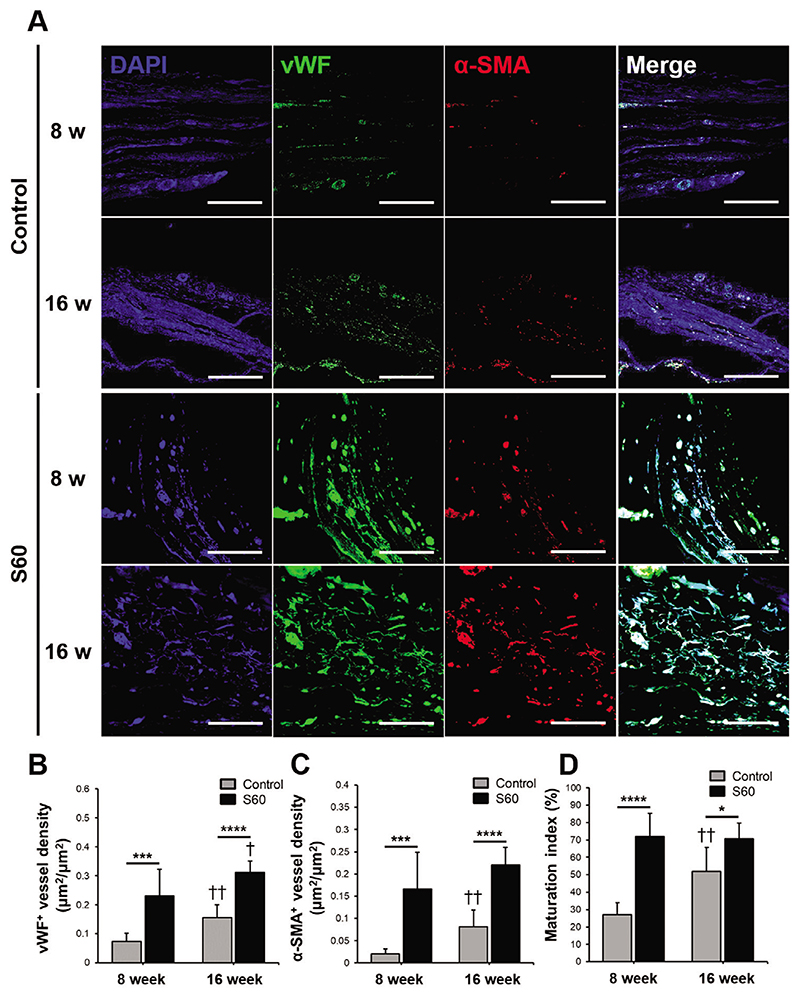
For sections through the center of the explanted scaffolds/defects: A) Representative immunofluorescence staining of control and S60 implanted calvarial defect at 8 and 16 weeks for angiogenic evaluations; vWF (green) and *α*-SMA (red) co-immunolabeling was used to assess the maturation of vessels (scale bars: 100 μm). Quantitative analysis ofB) vWF positive vessel density, C) *α*-SMA positive vessel density, and D) maturation index measured by ImageJ. The maturation index was based on comparison of the percentage of *α*-SMA positive vessel area in the total vessel area *(n =* 4, *P < 0.05, ***P < 0.0005 and ****P < 0.00005; †*P <* 0.05 and ††*P <* 0.005 compared with 8 weeks of the same group).

Angiogenesis and mature vessel formation within the calvarial defect site, with and without S60 scaffold implantation, were evaluated since angiogenesis and vascular tissue formation must be induced for bone regeneration. In order to confirm angiogenic activity and vessel maturation, histology sections were examined by double immunofluorescence staining of von Willebrand factor (vWF) and alpha smooth muscle actin *(a*-SMA) (Figure 6A). As shown in Figure 6B, vWF-positive cell density within a S60 implanted defect was threefold and twofold higher than that of the control group at 8 and 16 weeks post-surgery, respectively. This indicates that S60 scaffold enhanced new blood vessel formation and angiogenic activity. Additionally, *a*-SMA positive vessel density for S60 group was eightfold and threefold higher than that of the control group at 8 and 16 weeks after implantation, respectively (Figure 6C). The maturation index; based on double staining results (Figure 6D), confirmed that implanted S60 scaffold enhanced vessel maturation. For the control group, vessel density (vWF and *a*-SMA positive staining) and maturation index increased from 8 to 16 weeks post-surgery. On the other hand, S60 did not show significant differences between 8 and 16 weeks post implantation. The vessel density and maturation index values were higher than that of the control group at all the time points. Thus, the results imply that the S60 scaffold encourages angiogenesis and vessel maturation, starting from an early stage. This could be due to silica release from S60 scaffold, or the surface chemistry and 3D architecture. The degradation mechanism of sol-gel bioactive glasses is well established.^[9]^ Silica released from mesoporous silica microspheres was shown to up-regulate hypoxia inducing factor *1α* (HIF-1*α*) expression and stabilize the activation through down-regulating HIF-prolyl hydroxylase 2 in human umbilical vein endothelial cells culture. This process successively induced key angiogenic factors, such as basic fibroblast growth factor, vascular endothelial growth factor, and endothelial nitric oxide synthase,^[41]^ which could explain angiogenic characteristics of S60. Poly(MMA-*co*-TMSPMA)-SiO_2_ hybrids have been shown to release silica. For example, a similar inorganic:organic composition to our S60 hybrid lost mass over a period of 100 days degradation in PBS.^[17]^ However, the Si concentrations reported were 10 to 25 μg mL^-1^, which is higher than that released by our scaffolds (4.8 ± 0.1 μg mL^-1^ released over 16 weeks, Figure S2, Supporting Information). As the Si release is small, the surface chemistry and pore architecture are also likely to contribute to the angiogenic effect.

**Figure 7:**
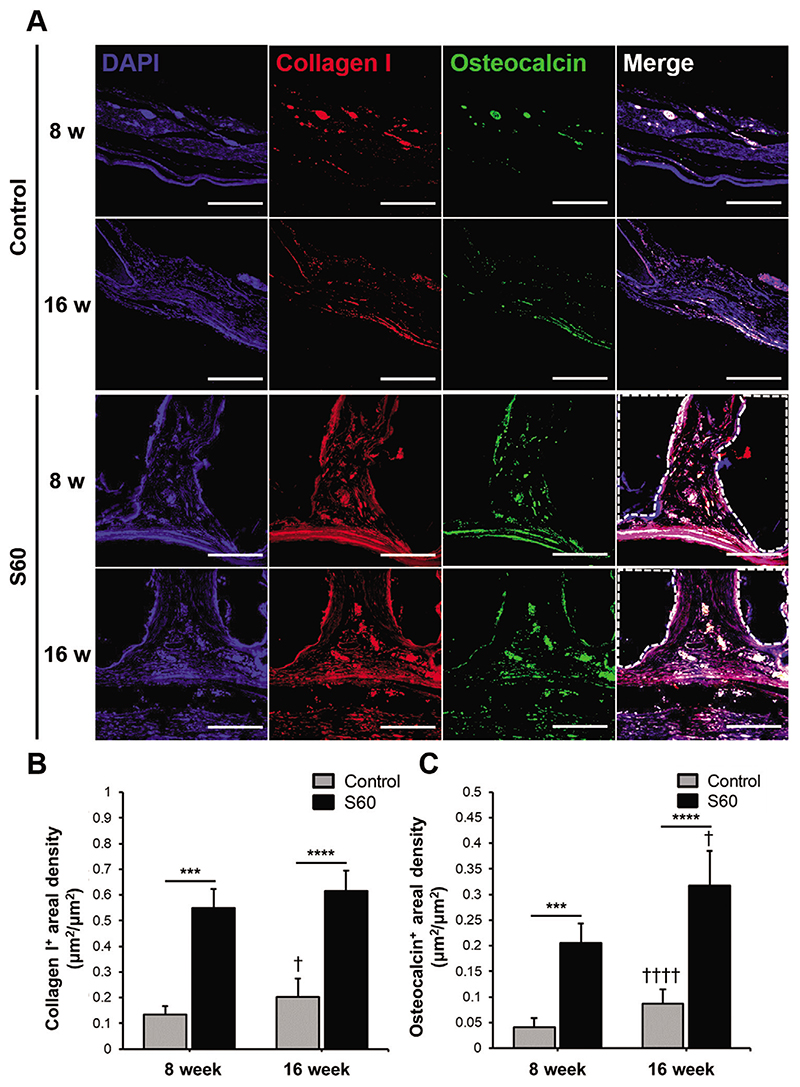
For sections through the center of the explanted scaffolds/defects: A) Immunofluorescence staining of collagen Type I and osteocalcin in control and S60 implanted calvarial defect at 8 and 16 weeks; collagen type I (red) and osteocalcin (green) co-immunolabeling was used to assess bone tissue regeneration. White dotted lines indicate the scaffold region (scale bars: 100 μm). Quantitative analysis of B) collagen type I positive staining areal density, and C) osteocalcin positive staining areal density measured by ImageJ. (*n* = 4, ***P < 0.0005 and ****P < 0.00005; †*P <* 0.05 and ††††*P <* 0.00005 compared with 8 weeks of the same group)

To verify osteogenic differentiation and bone production as a result of S60 scaffold implantation, immunostaining of osteogenic markers, that is, collagen Type I and osteocalcin, were evaluated (Figure 7A). Collagen Type I is one of the main components of bone that gives strength by forming oriented layers, while osteocalcin acts as an essential linker between the organic and inorganic components in the bone matrix. Both markers were more highly expressed in defects containing S60 scaffolds compared to that of the control group, as shown in Figure 7B,C, regardless of the post-implantation time points. The osteogenic markers confirmed that S60 enhanced bone regeneration, due to the release of silica species which stimulate collagen Type I and osteoblastic differentiation^[42]^,or a combination of 3D architecture and surface chemistry of the scaffold.

Overall, S60 was biocompatible and able to promote new vascularized bone tissue formation within the pore channels with the support of its surface, which promoted cell adhesion. Bone growth was starting in the central part of the defect area, that is, inside S60, which was not contiguous to calvarial defect edges. The combination of S60 scaffold induced both blood vessel and bone growth.

**Figure 8:**
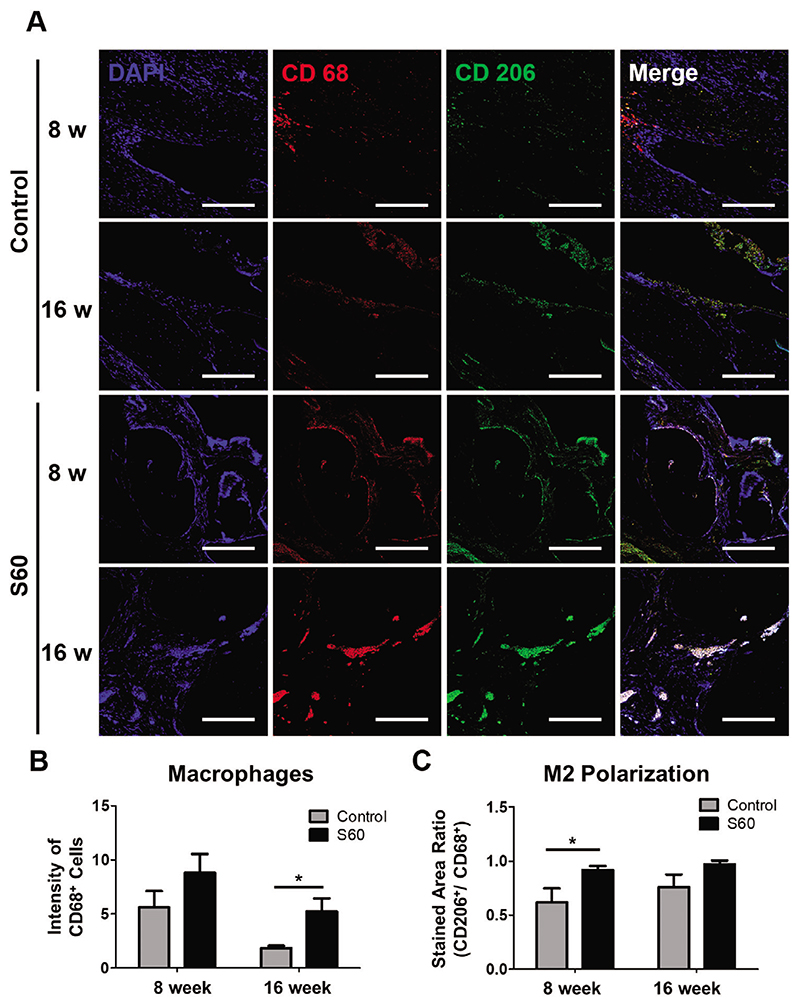
For sections through the center ofthe explanted scaffolds/ defects: A) Macrophage immunofluorescence staining in control and S60 implanted calvarial defect at 8 and 16 weeks. CD 68 (red) and CD 206 (green) co-immunolabeling was used to assess the phenotypic profiles of macrophages (scale bars: 200 μm). Quantitative analysis of B) CD 68 positive staining, and C) CD 206 to CD 68 ratio measured by ImageJ. (*n* = 4, *P < 0.05)

Macrophage polarization in the defect site and S60 at 8 and 16 weeks post-surgery were assessed, since macrophages are known to be involved in the bone healing process.^[43]^ Immunolabeling of CD68 (pan macrophage) and CD206 (M2 marker) was performed to identify the number, location, and phenotypic profiles of macrophages (Figure 8A). Generally, macrophages are known to polarize into two groups; M1 and M2, depending on the microenvironment. M1 macrophages act as a pro-inflammatory agent, while M2 macrophages repair damaged tissues. More macrophages were found in defects with S60 compared to the control group regardless of the time points (Figure 8B), which is not surprising, as a material was present. M2 macrophage ratio was also higher in the S60 samples compared to the control group (Figure 8C). Of note, M1 macrophages (CD 206 negative cells) appeared in the control group after 8 weeks post-surgery, although it is considered beyond the early stage of inflammation. It is well-known that M2 macrophages play crucial roles in promoting bone repair and improving biomaterial to tissue integration.^[44]^ A previous study was able to show that soluble silica released from bioactive glass stimulated macrophage polarization toward M2 phenotype, which concurs with the results from this study.^[45]^

## 3 Conclusion

Poly(MMA-*co*-TMSPMA)-*star*-SiO_2_ hybrid “ink” with three different inorganic:organic compositions were successfully 3D printed via direct ink writing. 3D printing allowed production of hybrid scaffolds with controlled interconnected porous structure with pore size in excess of 100 μm. S60 had the most ideal printability, while its porosity and mechanical properties fell within the range of trabecular bone. In vitro and in vivo studies confirmed vascularized bone regeneration ability. Additionally, S60 showed a preferable M1/M2 macrophage profile, which led to pro-healing microenvironment coupled with augmented osteogenesis. The authors believe that this study opens up more possibilities for hybrid materials to be applied toward bone repairing biomaterials.

## 4 Experimental Section

### Star Polymer Synthesis

MMA (99%), TMSPMA (98%), ethylene glycol dimethacrylate (EGDMA, 98%), cumyl dithiobenzoate (CDB, 98%), toluene (99%), and tetrahydrofuran (THF, 99.9%) were obtained from Sigma–Aldrich. Azobisisobutyronitrile (AIBN, 98%) was obtained from Molekula, and recrystallized in ethanol prior to the polymerization. *n*-hexane was obtained from Fisher Chemical.

Poly(MMA-*co*-TMSPMA)-*star* was synthesized using RAFT polymerization technique, following a same process as the previous study.^[20]^ Linear, or arm of the star polymer, poly(MMA-*co*-TMSPMA) was first synthesized with the molar ratio of MMA_100_-TMSPMA_10_ using toluene as the solvent.

AIBN was used as the initiator and CDB was used as the RAFT agent with a molar ratio of 1:2. The reagents were introduced in a round bottle flask in the overall molar ratio of AIBN_1_:RAFT_2_:MMA_480_:TMSPMA_48_. RAFT polymerization was performed at 70 °C in an argon atmosphere under continuous stirring and stopped at 50% conversion. The polymer was then precipitated in excess of *n*-hexane, and re-dissolved in toluene. The obtained polymer was dried using rotary evaporator to measure the mass of the polymer synthesized.

The arm of the star polymer was introduced in a round bottle flask with a bi-functional branching agent (EGDMA), AIBN, and toluene as a solvent in the molar ratio of ARM_1_:EGDMA_8_:AIBN_0.3_. The polymerization was performed at 70 °C in an argon atmosphere for 18 h under continuous stirring. The star polymer was then precipitated in excess of *n*-hexane and ethanol, and then dissolved in THF. The obtained polymer was dried using rotary evaporator to measure the mass ofthe polymer synthesized and re-dissolved in THF.

### Hybrid Ink Synthesis

Tetraethyl orthosilicate (TEOS, 98%) was obtained from ABCR GmbH & Co. KG, and 1_M_ hydrochloric acid (HCl) from Fisher Chemical.

Inorganic source of the hybrid was prepared by hydrolyzing TEOS with the molar ratio of TEOS_1_:water_3.7_:HCl_0.01_. The reagents were stirred vigorously for 40-60 min, full hydrolysis of TEOS was visually confirmed; from cloudy to clear. Then, the star polymer (immersed in THF, 50% v/v) was mixed with hydrolyzed TEOS according to three different inorganic:oganic wt% ratios of 50:50, 40:60, and 30:70. The mixture was left to continuously stir (40-60 min) until it was viscous enough to be printed.

The appropriate viscosity was determined visually and indicated by being able to draw thread of the hybrid gel from the bulk mixture using a syringe needle. The partially gelled hybrid ink was then transferred to luer lock syringe and aged at room temperature for another 40 min before printing.

### 3D Printing

A polyethylene tubing (Harvard Apparatus PolyE, internal diameter of 1.78 mm) was attached to the Luer lock syringe that contained the hybrid ink. At the opposite end of the tubing, polypropylene printing nozzle (Nordson EFD, SmoothFlow tapered tip, 0.2 mm) was connected. The syringe-tubing-nozzle was connected to the syringe pump (Harvard Apparatus PHD ULTRA) with constant applied force (flow rate of 0.04 ml s^-1^), and the nozzle was guided by a 3D printer (Ultimaker Original) with the control by computer aided direct-write program (Cura 13.10) at a speed of 10 mm s^-1^.The program was set upto print 9 mm × 9 mm × 8 mm with a 90° mesh architecture where each layer contained an array of parallel struts that were aligned perpendicular to the previous layer. The inter-strut distance was set to 0.5 mm. Each layer consisted of a total of 19 struts and each scaffold consisted of 40 layers. After printing was complete, the hybrid scaffolds were placed in a sealed Nalgene container and aged slowly at room temperature for 2 days and in a 40 °C oven for another 2 days. Then, the scaffolds were dried in 60 °C oven for one day.

Rokit Invivo 3D printer was used for fabricating 3D scaffolds for the in vivo (calvarial defect model) study. Printing ink, or sol stage hybrid mixture, was transferred to 10 mL (12mL) NORM-JECT Luer Lock syringe with Nordson EFD SmoothFlow tapered tip (0.2 mm). The syringe was placed in the Bio Dispenser and cylinder with 10 mm (diameter) × 2mm (height) was printed with NewCreatorK 3D printing software. The slicer setting was 0.1 mm layer height, 60% fill density, Grid infill pattern, 10 mm s^-1^ printing speed, 10 mm s^-1^ traveling speed, and 300% input flow.

### Hybrid Scaffold Characterization

The functional groups of the hybrid compositions were confirmed by Fourier transform infrared spectroscopy (Nicolet iS10, Thermo Scientific) with an attenuated total reflectance module set up. 32 scans were averaged to a 4 cm^-1^ resolution.

Inorganic–organic wt% ratios were analyzed by thermogravimetry analysis (Netzsch Sta 449c). The ground down samples were placed in a platinum crucible and heated to 800 °C at 10 °C min^-1^ with constant flow of air.

The printed hybrid scaffold’s microstructure was confirmed by scanning electron microscope (JEOL JSM-6010LA Analytical SEM). The electron beam voltage was accelerating at 20 keV with a spot size of 60. The working distance was set to 15 mm, and secondary electron imaging mode was used. Pore sizes were measured using ImageJ image processing software. 40 pores of each compositions’ horizontal and vertical cross-sections were measured using particle analysis function.

The skeletal volume and density of the scaffold was measured by gas pycnometer measurement (Ultrapycnometer 1000, Quantachrome Corporation). Four S60 samples were weighted and analyzed together for 10 repetitions.

The mechanical properties of the samples were confirmed by a uniaxial compression test (Zwick Roell Z2.5). The samples were loaded perpendicular to the force applied direction regarding their printed layers. 2 kN load cell was used, pre-load was set to 0.05 N, and the compression speed was 0.2 mm min^-1^. Three 3D printed hybrid scaffolds of each composition were analyzed. The test scaffolds were ground down to make surfaces flat and cube structure as possible. Their average dimensions were length: 4.9 ± 0.3 mm, width: 5.0 ± 0.3 mm, and height: 4.7 ± 0.3 mm. Cyclic compressive loading test was set up similar to the compression test, but the maximum force applied was limited to 12 MPa, and point of load removal cycle was set at standard travel of 0 mm with 60 s hold time. 10 cycles were performed on a ground down S60 hybrid scaffold with a dimension of 4.4 × 4.5 × 4.4 mm.

Dissolution study of S60 hybrid scaffold was confirmed by measuring silica release profile in phosphate-buffered saline (PBS, pH 7.4, 1X) solution. S60 scaffolds (*n* = 3) were immersed in PBS solution with a ratio of 30 mg inorganic content (Si content from the scaffold) to 20 mLof PBS in a Nalgene PMP jar. Dissolution containers were kept in an incubating orbital shaker (37 °C) and agitated at 110 rpm. The samples were incubated for 1, 2, and 4 weeks. At the end of each time point, the dissolution containers were removed from the incubator. 1 mL of the media was collected using a pipette and replaced by 1 mL of fresh PBS solution for compensation. The collected 1 mL sample solution was mixed with 9 mL of 2m nitric acid to prepare for inductively coupled plasma optical emission spectroscopy (ICP-OES, Thermo Scientific iCAP 6500 Duo) analysis. Si 1000 ppm ICP standard was prepared to 0.5, 1, 5, 10 ppm for the calibration curve.

### MC3T3-E1 Cell Culture

Reagents used for cell culture were purchased from Thermo Fisher Scientific and Sigma–Aldrich UK unless otherwise specified. MC3T3-E1 osteoblast precursor cell line (ATCC, UK) were monolayer expanded in basal media (*α*-MEM supplemented with 10% (v/v) FCS and 1% (v/v) penicillin-streptomycin) and maintained at 37 °C in 5% CO_2_ and humidified atmosphere until confluent. Cells were passaged using 500 μg mL^-1^ trypsin-EDTA for cytotoxicity and cell attachment assays.

### Preparation of Cytotoxicity Test Dissolution Products

The hybrid scaffolds in powder form were fabricated and used for the preparation of dissolution products following the guidelines within ISO10993-12^[46]^ (Biological evaluation of medical devices Part 12: Sample preparation and reference materials). Dissolution products released by the hybrid scaffolds at 0.2g mL^-1^ in *α*-MEM over 72-h at 37 °C were prepared. Dissolution products of medical grade polyethylene (PE) and polyurethane (PU) containing 0.1% (w/w) zinc diethyldithiocarbamate (ZDEC) were also prepared and served as non-cytotoxic negative control and cytotoxic positive control respectively. All dissolution products were sterilized through a membrane with 0.2 μm pores and, dilution series (25%, 50%, 75% and 100%) were prepared and supplemented with 10% (v/v) FCS prior to use in cytotoxicity assays.

### Cell Viability Analysis

Cellular viability in response to the dissolution products was tested by 3-(4,5-dimethylthiazol-2-yl)-2,5- diphenyltetrazolium bromide (MTT) assay in accordance to ISO10993- 5^[36]^ (Biological evaluation of medical devices Part 5: Tests for in vitro cytotoxicity). Briefly, 1 × 104 MC3T3-E1 cells were seeded onto each well in 96-well plates. Following 24 h of culture in basal *α*-MEM, cells were exposed to the dissolution products of hybrid scaffolds or controls for further 24 h. MTT powder was dissolved in plain *α*-MEM at the concentration level of 1 mg mL^-1^. After the incubation period, culture media was replaced by MTT solution. Cells were incubated for 2 h until purple formazan precipitates became visible. MTT solution was removed and DMSO was added to dissolve precipitates for 5 min. The optical density was measured spectrophotometrically at 570 nm using a microplate reader (SpectraMax M5).

### Cell Culture on 3D Printed Hybrid Scaffolds

Hybrid scaffolds (approximately 5 × 5 × 5 mm^3^) were manufactured and sterilized with 70% ethanol. Following washing with PBS, each sample was placed in serum-free *α-* MEM for 30 min prior to cell seeding. MC3T3-E1 cells were harvested and suspended in basal *α*-MEM at a concentration 1 × 107 cells mL^-1^. 10 μl of cell suspension was seeded onto each scaffold and incubated for 2 h with gent agitation every 20 min to ensure well distributed cell seeding. Each scaffold was then submerged in fresh basal *a*-MEM and cultured for further 24 h.

### Visualization and Quantification of Cell Attachment

Vimentin and Actin staining: Cell-seeded samples were washed in PBS and fixed in 4% (w/v) paraformaldehyde for 24 h. Following permeabilisation with buffered 0.5% Triton X-100 in PBS (300 mM sucrose, 50 mM NaCl, 3 mM MgCl_2_, 20 mM Hepes and pH 7.2), immunostaining involved a 5 min incubation in 1% (w/v) BSA followed by hour-long incubation in anti-Vimentin antisera (1:500 dilution in 10 mg mL^-1^ BSA in PBS, rabbit polyclonal, IgG, Ab- cam, Cambridge, UK) at 4 °C. Secondary anti-rabbit antibody (Alexa Fluor 488 conjugated, Abcam, Cambridge, UK) and Alexa Fluor 568-conjugated phalloidin (1:1000 dilution in 1% (w/v) BSA) was then added for 1 h incubation. DAPI (0.1μg ml^-1^ in PBS, 10 min incubation) was used as a nuclear counter stain. No staining was observed in isotope primary antibody control groups. Samples were imaged under confocal microscopy (Leica SP5 Laser Scanning Confocal Microscope and software, Leica Microsystems, Wetzlar, Germany).

Total DNA quantification: The total DNA content of MC3T3-E1 cells on each scaffold was determined using fluorescent Hoechst stain. Cells were lysed by freeze-thaw cycles following the removal of culture media. Lysed cells were stored at -80 °C and thawed to room temperature on the day of assay. The lysate was incubated with molecular biology grade water for 1 h and Hoechst stain was added at a final concentration of 2 μg ml^-1^. The fluorescence of Hoechst dye was measured at 360 nm excitation wavelength and 460 nm emission wavelength. DNA and MC3T3-E1 cell standard curves were obtained from serial dilutions of known DNA (calf thymus, Sigma, UK) concentrations and cell densities respectively. The percentage of cells attached on the scaffold = Total DNA content from cell-scaffold construct / Total DNA of seeded cells × 100%.

### Animals and Surgical Procedure

All animals (SD-RAT, male, 8 weeks, 250 ± 15 g; Samtako, Korea) were cared for according to the methods approved by the Institutional Animal Care and Use Committee at Korea Institute of Science and Technology (2017-105). For the surgery, temporary anaesthesia was performed by intramuscular injection with a cocktail of zolazepam and tiletamine (0.3 mL kg^-1^, Zoletil, Vibrac, France) and xylazine (0.1 mL g^-1^, Rompun, Bayer, Germany). 8 mm round calvarial defects were created with a trephine bur (TPHB-B8, OSUNG, Korea) following the periosteum removal. The prepared samples were implanted (*n* = 6) into the defect and the incision was closed with suture. For the control group, defects without scaffolds were used (*n* = 6). Each implant was analyzed after 8 and 16 weeks by micro-computed tomography (CT) and histological staining analysis.

### Micro-CT Analysis

Quantitative 3D analysis of the calvarial defect samples was conducted using a Skyscan 1172 CT (Bruker, USA). At 8, 16 weeks following implantation, the animals were sacrificed and the bone defects were harvested. The specimens were fixed in 10% (v/v) buffered neutral formalin for 1 day. Samples were securely in a 5-mL conical tube and centered in the micro-CT machine. After calibration, samples were scanned using the following settings: 49 kV, 200 μA, and Al 0.5 mm filter. Raw data were collected and reconstructed using the NRecon software (Bruker, USA) and the volume of newly formed bone was calculated using the DataViewer (Bruker, USA) and CTAn software (Bruker, USA).

### Histological Analysis

The specimens from each group were decalcified in a decalcifying solution (Sigma, USA) for 6 h, followed by serial dehydration with a graded ethanol series (80-100%). Then, the samples were embedded in paraffin and sectioned at 10 μm thickness and then stained with hematoxylin and Eosin (H&E) and Masson’s trichrome (MT) stains. The stained area was calculated with the MT-stained images (*n* = 4) using the ImageJ (National Institutes of Mental Health, USA).

### Immunofluorescent Analysis

The central regions of S60 and control group defects were characterized. The micro-sectioned specimens were stained with CD68 (ab955, Abcam, UK) and CD206 (sc-34577, Santa Cruz Biotechnology, USA) antibodies. Alexa fluor 594 anti-mouse IgG and Alex fluor 488 anti-goat IgG were used as secondary antibodies, respectively. The nuclei were counterstained with DAPI (Molecular probes, USA), and the stained sections were observed using confocal laser scanning microscopy (LSM700, Zeiss, Germany). For evaluating the density of vascular cells in the defect region, endothelial cells (ECs) and vascular smooth muscle cells (SMCs) were stained by polyclonal rabbit anti-human von Wille-brand factor antibody (vWF, sc-365712 FITC, Santa Cruz Biotech, USA) and monoclonal mouse anti-human *a*-smooth muscle actin antibody (*α*-SMA, sc-53142 AF594, Santa Cruz Biotech, USA), respectively. vWF and *a*-SMA- positive vessels in three fields were randomly selected and evaluated using ImageJ program (*n* = 3 in each group). The maturation index was quantified as the ratio of smooth muscle cell-positive vessels to total vessel area. Furthermore, to verify the osteogenesis, the samples were stained with type I collagen (ab34710, Abcam, USA) and osteocalcin (sc-390877 FITC, Santa Cruz Biotech, USA) antibodies. For quantifying bone tissue regeneration in the defect region, type I collagen and osteocalcin positive regions in three fields were randomly selected and evaluated using ImageJ program (*n* = 3 in each group). The type I collagen and osteocalcin positive areal density was quantified as the ratio of type I collagen and osteocalcin positive areas to total tissue area, respectively.

### Statistical Analysis

The statistical analysis was performed using one-way analysis of variance (ANOVA) with the Turkey’s significant difference post hoc test using SPSS software (IBM, USA). A value of *P <* 0.05 was considered as statistically significant.

## Supplementary Material

Supplemental Video 1

Supporting Information

## Figures and Tables

**Table 1:** Mechanical properties of the 3D printed hybrid scaffolds made with different organic contents in wt%: 70%, (S70); 60%, (S60); 50% (S50) (*n* = 4). Average horizontal and vertical cross-section pore sizes measured by SEM image analysis. Total DNA content of MC3T3-E1 cells on each scaffold determined by fluorescent Hoechst stain after 24 h of cell culture.

Scaffold	Young’s Modulus [MPa]	Ultimate stress [MPa]	Failure strain [%]	Horizontal cross-section pore size [μm]	Vertical cross-section pore size [μm]	Total DNA quantification [%]
S70	101 ± 17**	5.9 ± 1.3**	7.4 ± 0.2^*^	190 ± 49	111 ± 27	36 ± 7*
S60	247 ± 12**	22.4 ± 2.8	11.5 ± 2.0	191 ± 32	151 ± 29**	56 ± 8
S50	372 ± 25**	26.3 ± 0.8	8.9 ± 0.5	180 ± 29	121 ± 33	59 ± 7

Standard deviations are derived from the average values

* *P*< 0.05 (significant difference within the column values)

** *P*< 0.01 (significant difference within the column values)

## Data Availability

Research data are not shared.
