# Supplementary material for: 3D Printed Porous Methacrylate/Silica Hybrid Scaffold for Bone Substitution
Source: Adv Healthc Mater. Author manuscript; Available in PMC 2024 Jan 11. (PMC7615494; doi:10.1002/adhm.202100117)
Supplement: Supporting Information [file EMS124817-supplement-Supporting_Information.docx]

3D printed porous methacrylate/silica hybrid scaffold for bone substitution

Justin J. Chung^1,2†^, Jin Yoo^2†^, Brian S. T. Sum^1^, Siwei Li^1^, Soojin Lee^2^, Tae Hee Kim^2^, Zhenlun Li^1^, Molly M. Stevens^1,3,4^, Theoni K. Georgiou^1^, Youngmee Jung^2,5,6^*, Julian R. Jones^1^*

^1^Department of Materials, Imperial College London, SW7 2AZ, London, United Kingdom

^2^Center for Biomaterials, Biomedical Research Institute, Korea Institute of Science and Technology (KIST), Seoul, 02792, Republic of Korea

^3^Institute of Biomedical Engineering, Imperial College London, SW7 2AZ, London, United Kingdom

^4^Department of Bioengineering, Imperial College London, SW7 2AZ, London, United Kingdom

^5^School of Electrical and Electronic Engineering, Yonsei University, Seoul, 03722, Republic of Korea

^6^YU-KIST Convergence Research Institute, Seoul, 03722, Republic of Korea
^†^Equally contributed

*Corresponding authors

Supporting Information


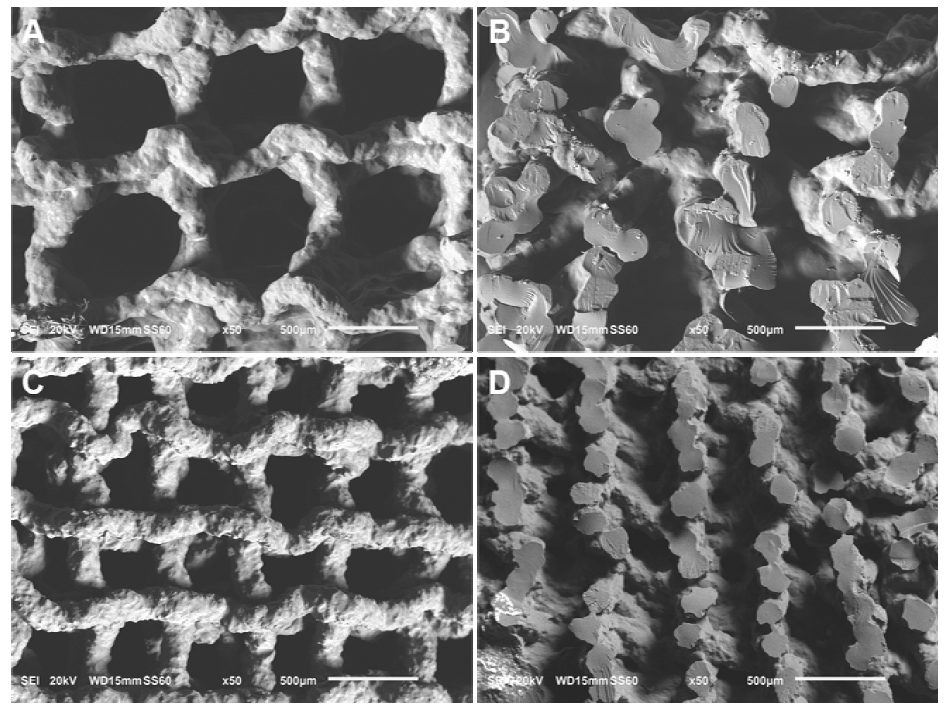


**Figure S1.** SEM images of 3D printed S60 scaffold with 1 mm inter-strut distance: A) top view, and B) vertical cross-section. SEM images of 3D printed S60 scaffold with 0.7 mm inter-strut distance: C) top view, and D) vertical cross-section.

**Figure S2.** Silicon dissolution profile of S60 hybrid scaffold upon immersion in PBS solution over 16 weeks.
