# Supplementary material for: 3D Printed Porous Methacrylate/Silica Hybrid Scaffold for Bone Substitution
Source: Adv Healthc Mater. 2021 May 5;10(12):2100117. doi: 10.1002/adhm.202100117 (PMC7615494; doi:10.1002/adhm.202100117)
Supplement: Supplementary file 1 [file ADHM-10-2100117-s001.pdf]

© 2021 Wiley-VCH GmbH

ADVANCED
HEALTHCARE
MATERIALS

Supporting Information

for *Adv. Healthcare Mater.*, DOI: 10.1002/adhm.202100117

3D printed porous methacrylate/silica hybrid scaffold for bone substitution

Justin J. Chung, Jin Yoo, Brian S. T. Sum, Siwei Li, Soojin Lee, Tae Hee Kim, Zhenlun Li, Molly M. Stevens, Theoni K. Georgiou, Youngmee Jung, Julian R. Jones**

#

3D printed porous methacrylate/silica hybrid scaffold for bone substitution

Justin J. Chung[1,2†], Jin Yoo[2†],Brian S. T. Sum[1], Siwei Li[1], Soojin Lee[2], Tae Hee Kim[2], Zhenlun Li[1], Molly M. Stevens[1,3,4], Theoni K. Georgiou[1], Youngmee Jung[2,5,6], Julian R. Jones[1]**

[1]Department of Materials, Imperial College London, SW7 2AZ, London, United Kingdom
[2]Center for Biomaterials, Biomedical Research Institute, Korea Institute of Science and Technology (KIST), Seoul, 02792, Republic of Korea
[3]Institute of Biomedical Engineering, Imperial College London, SW7 2AZ, London, United Kingdom
[4]Department of Bioengineering, Imperial College London, SW7 2AZ, London, United Kingdom
[5]School of Electrical and Electronic Engineering, Yonsei University, Seoul, 03722, Republic of Korea
[6]YU-KIST Institute,Yonsei University, Seoul, 03722, Republic of Korea
[†]Equally contributed
*Corresponding authors

Supporting Information

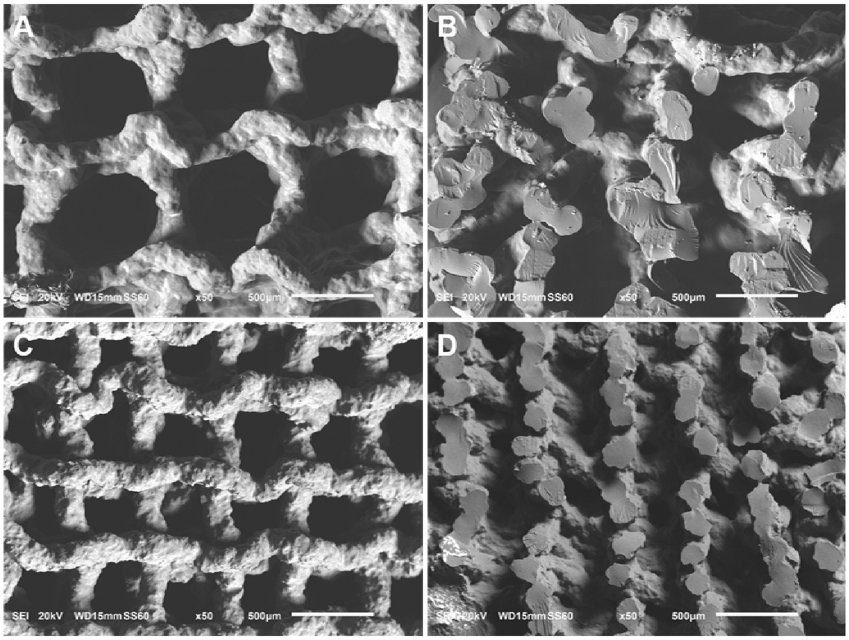

Figure S1: SEM images of 3D printed S60 scaffold with 1 mm inter-strut distance: A) top view, and B) vertical cross-section. SEM images of 3D printed S60 scaffold with 0.7 mm inter-strut distance:C) top view, and D) vertical cross-section.

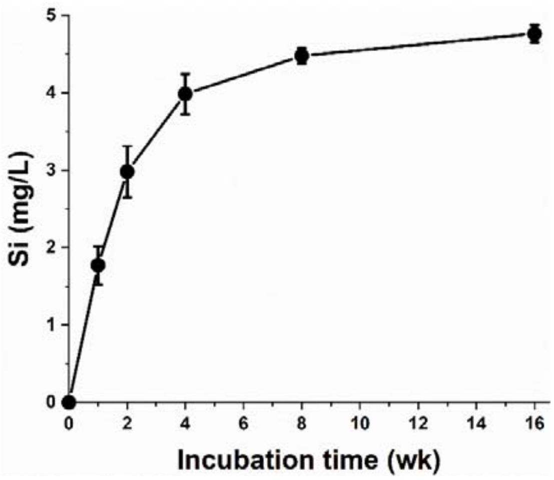

Figure S2: Silicon dissolution profile of S60 hybrid scaffold upon immersion in PBS solution over 16 weeks.
